# Impact of Dietary Patterns on Metabolic Syndrome in Young Adults: A Cross-Sectional Study

**DOI:** 10.3390/nu16172890

**Published:** 2024-08-29

**Authors:** Jingwen Liu, Wenfeng Lu, Qingyun Lv, Yaqi Wang, Xueying Xu, Yuan He, Hairong Chang, Yue Zhao, Xiaonan Zhang, Xiaoying Zang, Hua Zhang

**Affiliations:** School of Nursing, Tianjin Medical University, No. 22 Qixiangtai Road, Heping District, Tianjin 300070, China; liujingwen0929@163.com (J.L.); lwf980426@163.com (W.L.); 18722595311@163.com (Q.L.); wangyaqi1229@163.com (Y.W.); xuxueying2022@163.com (X.X.); heyuan1632@163.com (Y.H.); r1307375960@163.com (H.C.); yuezhao35@hotmail.com (Y.Z.)

**Keywords:** metabolic syndrome, dietary patterns, young adults, cross-sectional study, subgroup analysis

## Abstract

Metabolic syndrome has become a significant public health concern. This study aims to investigate the impact of dietary patterns on metabolic syndrome in young adults and how physical activity modulates this effect. A cross-sectional study was conducted at a health management center in Tianjin, China, from September 2022 to March 2023. Participants aged 18–35 years were recruited using convenience sampling. Dietary intake was assessed using a validated food frequency questionnaire. Logistic regression models evaluated associations between these patterns and metabolic syndrome, adjusting for potential confounders. Among 442 participants, four dietary patterns were identified: Legume–Nut, Alcohol–Meat, Sugar–Processed, and Egg–Vegetable. The Legume–Nut dietary pattern was associated with a higher risk of metabolic syndrome (OR = 2.63, 95% CI: 1.08–6.37), while the Egg–Vegetable dietary pattern was associated with a lower risk (OR = 0.26, 95% CI: 0.10–0.70). No significant associations were found for the Sugar–Processed and Alcohol–Meat patterns. Subgroup analysis revealed that the Legume–Nut pattern increased the risk of metabolic syndrome among those with irregular physical activity, whereas the Egg–Vegetable pattern decreased the risk. These findings highlight the significant influence of dietary patterns on the risk of metabolic syndrome in young adults and the modifying effect of regular physical activity, underscoring the need for targeted dietary and lifestyle interventions to prevent metabolic syndrome in this population.

## 1. Introduction

Metabolic syndrome is a clinical syndrome characterized by abdominal obesity, hypertriglyceridemia, low high-density lipoprotein cholesterol, elevated blood pressure, and increased fasting blood glucose [1]. Metabolic syndrome is a disease with severe public health consequences, reducing patients’ quality of life, increasing morbidity and mortality, and leading to substantial healthcare expenses [2]. The prevalence of metabolic syndrome in young populations has reached 17.4% [3], and its incidence is rising in both developed and developing countries [4]. However, current research on metabolic syndrome predominantly focuses on physiological, biochemical, clinical, and metabolic factors [5].

Existing research indicates that metabolic syndrome is closely associated with diet [6]. Specifically, carbohydrate intake is associated with metabolic syndrome, and reducing carbohydrate intake can be an effective treatment for metabolic syndrome [7]. Excessive sugar intake directly correlates with hyperglycemia and its complications [8]. Protein intake plays a crucial role in muscle protein synthesis and the maintenance of muscle mass [9], thereby impacting overall body metabolism [10]. Furthermore, diets high in fats, particularly excessive intake of saturated fatty acids, are closely linked to several core symptoms of metabolic syndrome [11].

In recent years, many studies have focused on the health status of young people, particularly metabolic syndrome [12,13]. However, most studies have not focused on dietary patterns, or the classifications of dietary patterns have been overly simplistic [14]. Studying dietary patterns offers a comprehensive assessment of the impact of food combinations on health, avoiding the biases that may arise from focusing solely on individual nutrients [15]. For instance, a study investigating the relationship between total sugar intake and metabolic syndrome in middle-aged individuals suggests that the focus should not solely be on the total sugar intake but rather on the contribution of sugar intake to the overall energy intake [16]. Furthermore, the impact of consuming isoenergetic amounts of sugar on health varies depending on the original form of the food, highlighting the limitations of focusing exclusively on individual nutrients. By analyzing overall dietary patterns, researchers can gain deeper insights into the complex interactions between different foods and their collective effects on metabolism, providing a scientific basis for effective public health strategies [17]. Another advantage of using dietary patterns in research is the ability to identify potential synergistic effects within the diet and their long-term impact on overall health [18]. Additionally, the research indicates that combining dietary therapy with physical activity can effectively improve the metabolic status in patients with metabolic syndrome [19]. Studying the health status of young people is particularly significant because their eating habits and lifestyles are relatively easier to modify. This makes them more amenable to interventions and facilitates the formulation of effective public health strategies.

Hence, this study aimed to explore the impact of dietary patterns on metabolic syndrome in young adults and how physical activity modulates this effect, providing new strategies for the prevention and treatment of metabolic syndrome.

## 2. Material and Methods

### 2.1. Data Sources and Study Population

**Figure 1:**
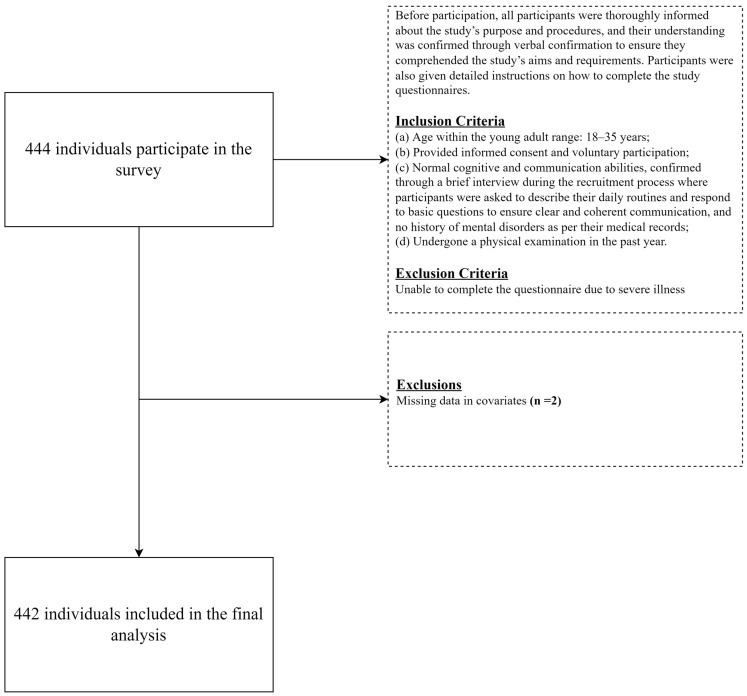
Flowchart of the sample selection process.

Participants were recruited using a convenience sampling method from a tertiary hospital’s health management center in Tianjin, China, from September 2022 to March 2023. Inclusion criteria were as follows: (a) age within the young adult range: 18–35 years; (b) provided informed consent and voluntary participation; (c) normal cognitive and communication abilities, confirmed through a brief interview during the recruitment process where participants were asked to describe their daily routines and respond to basic questions to ensure clear and coherent communication, and no history of mental disorders as per their medical records; (d) underwent a physical examination in the past year. Exclusion criteria included the inability to complete the questionnaire due to severe illness. Before participation, all participants were thoroughly informed about the study’s purpose and procedures, and their understanding was confirmed through verbal confirmation to ensure they comprehended the study’s aims and requirements. Participants were also given detailed instructions on how to complete the study questionnaires. Each questionnaire took approximately 25–30 min to complete. A total of 442 samples were included in this study (Figure 1).

### 2.2. Metabolic Syndrome

Metabolic syndrome is diagnosed when any three of the following criteria are present [20]: (1) increased waist circumference, with standards varying by population and country (≥90 cm for Chinese men, ≥85 cm for women) [21]; (2) elevated triglyceride levels: over 150 mg/dL (1.7 mmol/L), or under treatment for reduced triglycerides; (3) reduced HDL cholesterol: below 50 mg/dL (1.3 mmol/L) or under treatment to increase HDL-C; (4) elevated blood pressure: systolic ≥130 mmHg and/or diastolic ≥85 mmHg, or history of hypertension under treatment; (5) raised fasting blood glucose: ≥100 mg/dL (5.6 mmol/L), or history of diabetes under treatment. 

The disease is self-reported by the participant, or detected during a physical examination in the last year, or determined with relevant therapeutic medications. All diseases are diagnosed by a doctor, and all anthropometric measurements are con-ducted by nurses according to standard procedures.

### 2.3. Food Frequency Questionnaire (FFQ)

Dietary information was assessed using a validated semi-quantitative food frequency questionnaire (FFQ) [22,23,24], which has been widely employed in various studies [25]. Considering the consumption volume and frequency of food items among the surveyed population, the food items were selected based on the nutritional composition and dietary habits of Chinese individuals. This study included 17 major food groups and 189 food items within these groups, encompassing whole grains, tubers, beans and bean products, nuts, fresh vegetables, fruits, white meat, red meat, processed meat, aquatic and seafood products, milk and dairy products, eggs, cooking oils, fried food, sugary beverages, alcoholic beverages, and pastries (Supplementary Material Table S3). Participants were required to recall their frequency of intake and portion size consumed during the past 12 months.

Frequency options include: (1) every day; (2) 4–6 times a week; (3) 1–3 times a week; (4) 1–3 times a month; (5) almost never/never. During face-to-face interviewing, the portion size of each food item was estimated using food models and standard serving sizes (e.g., one standardized portion of cooked rice is one small bowl, weighing approximately 100 g). Then, the frequency of intake and portion size were used to calculate the amount of each food item consumed on average. These data were converted to grams or milliliters per day. Food intake was normalized for each participant. This study was normalized using a z-score, which is food intake minus the mean divided by the standard deviation. Finally, we calculated the factor scores of each participant for each pattern by summing the intake of food groups weighted by their factor loadings and grouped them into quartiles for further analysis, with Q1 corresponding to the lowest quartile of dietary pattern score. A higher quartile indicates more consistency with the pattern being calculated. Total energy intake was estimated through the FFQ. The intake of energy was calculated by using the China Food Composition Database (Version 6) [26].

### 2.4. Physical Activity

The World Health Organization recommends that for young adults, the most suitable physical activity should meet the following requirements: Heart rate should be maintained at 50–85% of maximum heart rate [27]. During moderate-intensity exercise, breathing should increase but still allow for conversation, while during high-intensity exercise, breathing should become rapid and conversation difficult, with a perceived exertion of 5–8 on a 10-point scale. Post-exercise, muscles should feel adequately fatigued but not overly sore, and joints should be free from abnormal pain. Body temperature should rise moderately with increased sweating. Each exercise session should last at least 30 min. Throughout and following the exercise, individuals should feel pleasant and satisfied, with adequate hydration and timely nutritional supplementation. The entire process should be safe and free from injury. In this study, the frequency of physical activity is categorized as monthly or less, weekly, 2–3 times per week, and 4+ times per week. ‘Monthly or less’ is defined as irregular physical activity, while ‘Weekly’, ‘2–3 times per week’, and ‘4+ times per week’ are defined as regular physical activity.

### 2.5. Statistical Analysis

Initially, the suitability of using factor analysis is determined by the Kaiser–Meyer–Olkin (KMO) measure and Bartlett’s test of sphericity. The results of the KMO test yielded a value of 0.771, and Bartlett’s test of sphericity showed a *p*-value < 0.001, indicating that factor analysis is appropriate for this study (Supplementary Material Table S1). Based on the correlation matrix and orthogonal varimax rotation, dietary patterns were derived. Eigenvalues greater than 1 were considered, and the scree plot indicated that the eigenvalues began to level off after the fourth principal component, contributing a smaller proportion of the total variance. Taking into account the specifics of this study, four factors were retained, extracting four distinct dietary patterns, with a cumulative variance contribution rate of 54.457% (Supplementary Materials Table S2 and Figure S1).

To present participant characteristics, continuous variables were expressed as mean values with standard deviations (SD), and categorical variables were presented as number and percentages. Characteristics between groups were compared using Chi-square tests, *t*-test, analysis of variance (ANOVA), or the Kruskal–Wallis rank sum test, as appropriate. To assess the relationship between dietary patterns and the incidence of metabolic syndrome, logistic regression models were established, providing odds ratios (OR) with their 95% confidence intervals (95% CI). Model 1 is an unadjusted crude model. Model 2 includes adjustments for age, gender, residence, education level, occupation, and marital status, while Model 3 further adjusts for age, gender, residence, education level, occupation, marital status, smoking, alcohol consumption, exercise frequency, sleep duration, height, weight and total energy intake (kcal/d). Subgroup analysis was performed according to physical activity frequency and also performed to detect potential modifiers.

Statistical analysis was carried out using IBM Statistical Package SPSS version 26.0 (SPSS Inc., Chicago, IL, USA) and R version 4.3.1 Forest plots were generated using the ‘forestplot’ package. Statistical significance in the analysis was indicated by a two-tailed *p*-value of less than 0.05.

This study was carried out in accordance with the Checklist for Strengthening The Reporting of Observational Studies in Epidemiology (STROBE), and it was approved by the medical ethics committee of Tianjin Medical University in China (TMuhMEC2022021) in 29 August 2022.

Each participant was given a written consent form before involvement in the study. The participants were briefed about the schedule, venue, and duration of data collection, along with the potential advantages and risks associated with their participation. Post obtaining informed consent, the principal investigator gathered data from both the medical records and the participants themselves. This methodology was followed until the targeted sample size was accomplished. The lead researcher meticulously read out the questions to all participants.

## 3. Results

### 3.1. Establishment of Dietary Patterns

This study identified four dietary factors through factor analysis, with factor load distributions shown in Table 1. Foods with a factor loading absolute value greater than 0.4 were considered to have a significant relationship with the respective factor. We independently named the dietary patterns based on their included food types, resulting in the following names: Legume–Nut dietary pattern, Alcohol–Meat dietary pattern, Sugar–Processed dietary pattern, and Egg–Vegetable dietary pattern. Each dietary pattern includes the following types of food as shown in Table 1.

### 3.2. Characteristics of Participants Grouped by Dietary Patterns

In this study, the average age of the participants was 24.79 ± 4.97 years, with a distribution showing more males (54.52%) than females (45.48%). Most participants resided in urban areas (70.14%), had completed a bachelor’s degree or higher (53.17%), and were unmarried (72.40%). A significant portion of the participants were non-smokers (75.11%) and non-drinkers (46.60%). Most of the participants were employed as mental workers (38.01%), and the majority exercised weekly (23.98%) (Table 2). The characteristics of study participants across quartile categories of the dietary pattern scores were shown in Table 3. Participants in the highest quartile (Q4) of the Legume–Nut dietary pattern were notably younger, predominantly male, taller, and heavier compared to those in the lowest quartile (Q1). They also were more likely to reside in rural areas and engage in more frequent physical activity. In contrast, individuals in the highest quartile of the Egg–Vegetable dietary pattern tended to be older, more likely to be male, taller, and heavier, with a higher likelihood of being married, smoking, and drinking. Additionally, those in the highest quartile of the Sugar–Processed dietary pattern were heavier, with higher rates of smoking and drinking, and engaged in less frequent physical activity. For the Alcohol–Meat dietary pattern, individuals in the highest quartile were older, predominantly male, taller, heavier, and more likely to smoke and drink.

### 3.3. Relationship between Dietary Patterns and Metabolic Syndrome

Table 4 outlines the relationship between dietary patterns and metabolic syndrome, presenting the results of the logistic regression analyses of the associations between various dietary patterns and metabolic syndrome. We constructed three covariate models to elucidate these associations, each with different levels of adjustment. After adjusting for multiple covariates, the fully adjusted logistic regression model indicates that participants in the top quartile of the Legume–Nut pattern scores had greater OR for metabolic syndrome (OR = 2.63; 95%CI: 1.08–6.37; *p* = 0.033) than those in the bottom quartile. Compared with those in the bottom quartile, participants in the top quartile of the Egg–Vegetable pattern scores had a lower OR for metabolic syndrome (OR = 0.26; 95%CI: 0.10–0.70; *p* = 0.007). In addition, no significant impact was observed of the Sugar–Processed dietary pattern on metabolic syndrome, nor of the Alcohol–Meat dietary pattern on metabolic syndrome (*p* > 0.05).

### 3.4. Subgroup Analysis in Physical Activity Frequency and Metabolic Syndrome

**Figure 2:**
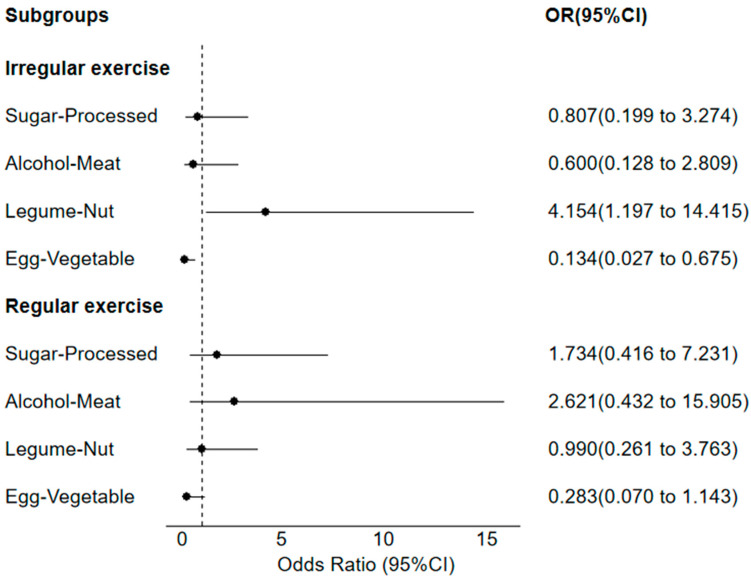
Subgroup analysis based on physical activity frequency.

As shown in Figure 2, within the irregular physical activity group, participants in the top quartile of the Legume–Nut pattern scores had greater OR for metabolic syndrome (OR = 4.15; 95%CI: 1.20–14.42; *p* = 0.025) than those in the bottom quartile. Compared with those in the bottom quartile, participants in the top quartile of the Egg–Vegetable pattern scores had a lower OR for metabolic syndrome (OR = 0.13; 95%CI: 0.03–0.68; *p* = 0.015). In addition, no significant association was observed between Sugar–Processed and Alcohol–Meat patterns and metabolic syndrome (*p* > 0.05). In the regular physical activity group, no significant association was observed between dietary patterns and metabolic syndrome (*p* > 0.05).

## 4. Discussion

This study examined the impact of dietary patterns and exercise frequency on metabolic syndrome among younger demographics. Four dietary patterns were identified: the Legume–Nut dietary pattern, the Alcohol–Meat dietary pattern, the Sugar–Processed dietary pattern, and the Egg–Vegetable dietary pattern. The findings revealed that the Legume–Nut dietary pattern is associated with a higher risk of metabolic syndrome, whereas the Egg–Vegetable dietary pattern is associated with a lower risk. The Sugar–Processed dietary pattern and the Alcohol–Meat dietary pattern did not show a significant association with the risk of metabolic syndrome. In the subgroup analyses, similar results were observed among young adults with irregular physical activity. However, among young adults with regular physical activity, no significant association was observed between dietary patterns and metabolic syndrome.

This study identified four major dietary patterns: the Sugar–Processed dietary pattern, characterized by high consumption of sugary beverages, pastries, and fried foods, featuring high levels of carbohydrates (especially refined sugars) and unhealthy fats; the Alcohol–Meat dietary pattern, primarily consisting of red meat, processed meats, and alcoholic beverages, noted for its high protein and fat content, particularly saturated fats and alcohol; the Legume–Nut dietary pattern, which includes significant intake of beans and bean products, nuts, and aquatic and seafood, rich in plant proteins, unsaturated fats, fibers, and micronutrients; and the Egg–Vegetable dietary pattern, marked by the high consumption of eggs, fresh vegetables, whole grains, and white meat, offering high-quality proteins, low fats, abundant fibers, vitamins, minerals, and carbohydrates. Research by the Chinese Center for Disease Control and Prevention’s Nutrition and Health Institute on dietary patterns among Chinese youth aged 18–35 indicates that these patterns can be categorized into the Traditional Rice dietary pattern, the Traditional Pasta dietary pattern and the High-Quality Protein dietary pattern [28]. These three dietary patterns closely resemble the Egg–Vegetable dietary pattern, the Legume–Nut dietary pattern, and Alcohol–Meat dietary pattern identified in this study. However, the earlier research did not include fried foods, sugary beverages, and pastries, which in our study, constituted the Sugar–Processed dietary pattern.

In the course of investigating the relationship between dietary patterns and metabolic syndrome, our findings indicate that the Legume–Nut dietary pattern is linked to an increased risk of developing metabolic syndrome. Conversely, the Egg–Vegetable dietary pattern appears to be associated with a reduced risk of metabolic syndrome. The Legume–Nut dietary pattern encompasses a variety of foods including tubers, beans, nuts, fruits, white meat, aquatic products, and seafood. This diet is predominantly characterized by a high intake of carbohydrates and sugars, particularly from potatoes and certain legumes that naturally contain significant amounts of starches and sugars [29,30]. In the absence of sufficient dietary fiber to mitigate these effects, there is an elevated risk of rapid blood sugar spikes [31]. Although fruits are abundant in natural fructose [32], excessive consumption can also result in fluctuations in blood sugar levels [33]. Nuts and seafood, while providing beneficial unsaturated fats that support cardiovascular health [34,35], can contribute to weight gain and negatively impact metabolic health if consumed in excess without adequate physical activity [36]. Furthermore, processed white meat and seafood may contain elevated levels of sodium [37], posing potential risks to blood pressure regulation and thereby increasing the likelihood of metabolic syndrome [38]. The Egg–Vegetable dietary pattern appears to be associated with a reduced risk of metabolic syndrome. This finding is consistent with a longitudinal study conducted in Australia, which examined the dietary patterns of young adults and their risk of metabolic syndrome and insulin resistance [14]. The Australian study found that a dietary pattern rich in fruits, vegetables, and whole grains was associated with a lower risk of metabolic syndrome. Similarly, our Egg–Vegetable dietary pattern is primarily composed of whole grains, fresh vegetables, eggs, and cooking oils, further supporting the beneficial effects of such diets on metabolic health. The high fiber content present in whole grains and fresh vegetables contributes to slower digestion and absorption, which in turn stabilizes blood sugar levels and promotes a prolonged sense of satiety, thereby preventing the overconsumption of high-calorie foods [39]. Eggs serve as a valuable source of protein, essential for muscle maintenance and overall metabolic health [40]. Additionally, healthy cooking oils, such as olive oil, provide essential monounsaturated fatty acids and small quantities of polyunsaturated fatty acids, both of which support normal cholesterol levels and cardiovascular health [41]. Furthermore, the low sugar content inherent in this dietary pattern aids in the prevention of metabolic disorders, including insulin resistance and diabetes [42].The Sugar–Processed dietary pattern and the Alcohol–Meat dietary pattern did not show a significant association with the risk of metabolic syndrome. This lack of association may be attributed to the relatively simplistic composition of these dietary patterns and the insufficient diversity of nutrient intake, which limits data variability. Furthermore, the absence of potential synergistic or interactive effects between foods may render the Sugar–Processed dietary pattern and the Alcohol–Meat dietary pattern less influential on metabolic syndrome compared to the Legume–Nut dietary pattern and the Egg–Vegetable dietary pattern, which are more nutritionally comprehensive and feature a greater variety of foods. Therefore, single or simplified food combinations may not be sufficiently relevant in studies addressing complex health conditions such as metabolic syndrome [17].

Subsequent subgroup analyses revealed that among individuals with irregular physical activity, the Legume–Nut dietary pattern is associated with a higher risk of metabolic syndrome, whereas the Egg–Vegetable dietary pattern is associated with a lower risk. In populations with irregular physical activity, the basal metabolic rate tends to be relatively low due to insufficient physical exertion, leading to reduced energy expenditure [43]. This condition makes the high-carbohydrate and high-fat content of the Legume–Nut pattern prone to causing an energy surplus and weight gain, further exacerbating the risk of metabolic syndrome [44]. Additionally, irregular physical activity may result in decreased insulin sensitivity and impaired blood glucose regulation [45]. Conversely, the Egg–Vegetable dietary pattern, characterized by its high fiber and low sugar content, can help improve blood glucose and insulin responses [46]. Thus, among those with irregular physical activity, this dietary pattern can effectively reduce the risk of metabolic syndrome. For individuals who engage in regular physical activity, the various physiological benefits conferred by physical activity, including an increased basal metabolic rate and improved glucose uptake by muscles and insulin sensitivity [45,47,48], may offset the potential negative impacts of different dietary patterns on the risk of metabolic syndrome. Furthermore, regular physical activity exhibits anti-inflammatory and antioxidant effects, which can effectively mitigate chronic inflammation and oxidative stress induced by unhealthy diets [49]. Consequently, the influence of dietary patterns on metabolic syndrome risk may be less pronounced in individuals who engage in regular physical activity. These findings underscore the importance of regular physical activity in preventing metabolic syndrome, demonstrating its efficacy across diverse dietary contexts.

The findings of this study highlight the crucial role of dietary patterns in preventing metabolic syndrome among young adults. Specifically, young adults, particularly those who do not engage in regular physical activity, should opt for high-fiber, low-sugar dietary patterns such as the Egg–Vegetable dietary pattern and avoid high-fat, high-carbohydrate dietary patterns like the Legume–Nut dietary pattern. This approach can aid in weight management, improve blood sugar regulation, and ultimately reduce the risk of metabolic syndrome. By adopting these healthier dietary habits and incorporating regular physical activity, young adults can enhance their overall metabolic health, decrease the likelihood of developing chronic conditions, and promote long-term well-being.

Nevertheless, it is crucial to recognize the specific limitations of this study. First, the sample size of this research is relatively small. This constraint may lead to insufficient statistical power, potentially affecting the generalizability and extendibility of the findings. Second, this cross-sectional study effectively reflects the status at a specific point in time but does not establish temporal sequence or causality, which may limit causal inference. Future research should include a larger sample size and employ a longitudinal design to track changes over time within the same cohort, thereby better assessing causal relationships and long-term effects. Additionally, this study may not have completely controlled for all potential confounding variables, such as socioeconomic status and genetic factors, which could influence the interpretation of the results.

## 5. Conclusions

In summary, this study revealed a significant association between dietary patterns and the risk of metabolic syndrome among younger demographics. These findings underscore the importance of balanced dietary choices as preventive strategies against metabolic syndrome. Future research should consider a larger sample size and a longitudinal design to better ascertain causal relationships and long-term effects. This approach can provide more definitive guidance for dietary recommendations to improve metabolic health in young adults.

## Figures and Tables

**Table 1:** Factor load in food groups of dietary patterns.

	Sugar–Processed	Alcohol–Meat	Legume–Nut	Egg–Vegetable
Whole Grains	−0.053	0.124	−0.007	0.439
Tubers	0.042	−0.057	0.729	−0.037
Beans and Bean Products	−0.080	−0.001	0.787	−0.019
Nuts	0.158	0.181	0.678	0.002
Fresh Vegetables	−0.102	−0.483	0.334	0.483
Fruits	0.208	−0.326	0.504	0.238
White Meat	0.042	−0.033	0.623	0.276
Red Meat	0.121	0.517	0.277	0.420
Processed Meats	0.446	0.503	0.343	0.058
Aquatic and Seafood	0.207	0.304	0.536	−0.025
Milk and Dairy Products	0.376	−0.547	0.170	0.309
Eggs	0.069	−0.068	0.144	0.706
Cooking Oils	0.134	0.037	−0.09	0.753
Fried Foods	0.566	0.560	0.106	0.100
Sugary Beverages	0.836	0.072	0.054	−0.006
Alcoholic Beverages	0.139	0.765	0.028	0.161
Pastries	0.791	0.044	0.077	0.048

Factor loadings of food groups in each dietary pattern were identified using factor analysis. The color gradation denotes the strength and direction of the correlation between the food groups and dietary patterns. Dark green indicates a relatively high correlation between the food group and the corresponding dietary pattern, with factor loadings greater than or equal to 0.4. Light green signifies a moderate correlation, with factor loadings less than 0.4 but greater than or equal to 0.2. Orange denotes a relatively low correlation, with factor loadings less than 0.2. The type of food with a black border is the composition of the food group.

**Table 2:** Characteristics of participants, Tianjin, China, 2022–2023.

Characteristics	Total	MetS− *N* (%) or M ± SD	MetS+ *N* (%) or M ± SD	*p* *χ*^2^ or *t*
Sample size (N)	442	361	81	
Age (years)	24.79 ± 4.97	24.53 ± 4.75	25.95 ± 5.74	0.041
Gender				0.344
Male	241 (54.52)	193	48	
Female	201 (45.48)	168	33	
Height (cm)	171.97 ± 8.50	171.98 ± 8.33	171.93 ± 9.26	0.958
Weight (kg)	66.12 ± 14.07	64.64 ± 13.17	72.70 ± 16.06	<0.001
Waist circumference (cm)	77.56 ± 12.77	74.58 ± 10.75	90.83 ± 12.68	<0.001
Hypertension				<0.001
No	363 (82.13)	358	5	
Yes	79 (17.87)	3	76	
Diabetes				<0.001
No	377 (85.29)	354	23	
Yes	65 (14.71)	7	58	
Dyslipidemia				<0.001
No	380 (85.97)	357	23	
Yes	62 (14.03)	4	58	
Residence				0.196
Urban area	310 (70.14)	258	52	
Suburban area	132 (29.86)	103	29	
Educational level completed				0.012
Junior middle school or below	113 (25.57)	85	28	
High school or Vocational school	94 (21.27)	72	22	
Bachelor’s degree or Junior college or above	235 (53.17)	204	31	
Marital status				0.017
Unmarried	320 (72.40)	270	50	
Married	122 (27.60)	91	31	
Smoking status				0.012
No	332 (75.11)	280	52	
Yes	110 (24.89)	81	29	
Drinking status				0.666
No	206 (46.60)	170	36	
Yes	236 (53.39)	191	45	
Occupation type				0.042
Physical workers	168 (38.01)	135	33	
Mental workers	60 (13.57)	43	17	
Students	214 (48.42)	183	31	
Sleep duration (hours/day)	7.27 ± 0.82	7.47 ± 0.90	7.38 ± 1.05	0.452
Physical activity frequency				0.005
Monthly or less	202 (45.70)	152	50	
Weekly	106 (23.98)	97	9	
2–3 times per week	95 (21.49)	80	15	
4+ times per week	39 (8.82)	32	7	

Statistical tests: Continuous variables were analyzed using independent *t*-tests or Welch’s *t*-test. Categorical variables were analyzed using Chi-square tests. MetS− indicates no metabolic syndrome. MetS+ indicates the presence of metabolic syndrome.

**Table 3:** Characteristics of participants grouped by dietary patterns.

Characteristics	*N*	Sugar-Processed		*p*	Alcohol-Meat		*p*	Legume-Nut		*p*	Egg-Vegetable		*p*
		Q1 (*n* = 111)	Q4 (*n* = 110)		Q1 (*n* = 111)	Q4 (*n* = 110)		Q1 (*n* = 111)	Q4 (*n* = 110)		Q1 (*n* = 111)	Q4 (*n* = 110)	
Age (years)	24.79 ± 4.97	25.41 ± 4.78	24.51 ± 5.32	0.100	23.42 ± 4.77	26.15 ± 4.82	0.001	24.28 ± 4.89	23.99 ± 4.73	0.033	21.86 ± 3.79	25.61 ± 5.01	<0.001
Gender				0.215			<0.001			0.006			<0.001
Male	241	55	65		26	87		49	67		27	84	
Female	201	56	45		85	23		62	43		84	26	
Height (cm)	171.97 ± 8.50	171.49 ± 8.24	171.78 ± 8.47	0.708	167.26 ± 7.75	174.87 ± 6.78	<0.001	169.28 ± 8.67	174.51 ± 7.52	<0.001	166.93 ± 6.76	175.67 ± 8.08	<0.001
Weight (kg)	66.12 ± 14.07	62.37 ± 13.74	71.16 ± 15.20	<0.001	58.10 ± 14.63	75.80 ± 12.66	<0.001	63.64 ± 14.82	68.89 ± 14.83	0.039	57.06 ± 12.32	74.39 ± 13.19	<0.001
Waist circumference (cm)	77.56 ± 12.77	75.41 ± 10.02	81.42 ± 16.75	0.002	72.92 ± 11.72	82.30 ± 15.69	<0.001	77.00 ± 11.87	76.37 ± 15.23	0.339	72.83 ± 11.65	79.25 ± 15.21	<0.001
Hypertension				0.028			0.110			0.131			0.750
No	363	96	80		97	83		99	87		94	87	
Yes	79	15	30		14	27		12	23		17	23	
Diabetes				0.767			0.134			0.919			0.896
No	377	96	91		100	90		96	92		95	96	
Yes	65	15	19		11	20		15	18		16	14	
Dyslipidemia				0.010			0.407			0.361			0.488
No	380	103	85		99	91		100	92		91	94	
Yes	62	8	25		12	19		11	18		20	16	
Residence				0.740			0.821			0.034			0.901
Urban area	310	80	75		77	74		80	68		80	77	
Suburban area	132	31	35		34	36		31	42		31	33	
Educational level completed				0.004			0.498			0.046			0.446
Junior middle school or below	168	15	35		23	30		29	20		22	27	
High school or Vocational school	60	25	26		30	21		28	23		20	25	
Bachelor's degree or Junior college or above	214	71	49		58	59		54	67		69	58	
Marital status				0.565			0.065			0.363			<0.001
Unmarried	320	76	81		86	70		83	84		101	77	
Married	122	35	29		25	40		28	26		10	33	
Smoking status				0.037			<0.001			0.220			<0.001
No	332	90	80		102	55		88	86		102	74	
Yes	110	21	30		9	55		23	24		9	36	
Drinking status				0.050			<0.001			0.173			<0.001
No	206	60	45		75	25		57	56		83	42	
Yes	236	51	65		36	85		54	54		28	68	
Occupation type				0.445			0.098			0.006			<0.001
Physical workers	168	40	43		33	49		41	30		25	43	
Mental workers	60	18	17		14	16		10	16		8	17	
Students	214	53	50		64	45		60	64		78	50	
Sleep duration (hours/day)	7.45 ± 0.93	7.62 ± 0.79	7.28 ± 1.07	0.055	7.57 ± 0.87	7.31 ± 1.07	0.208	7.42 ± 0.92	7.47 ± 1.19	0.879	7.60 ± 1.14	7.42 ± 0.88	0.323
Physical activity frequency				0.004			0.268			0.005			0.073
Monthly or less	202	40	63		44	54		56	41		40	50	
Weekly	106	30	15		30	22		28	20		33	20	
2-3 times per week	95	26	22		30	20		23	29		31	26	
4+ times per week	39	15	10		7	14		4	20		7	14	
Metabolism Syndrome				0.023			0.071			0.125			0.996
No	361	16	31		97	82		99	86		91	89	
Yes	81	95	79		14	28		12	24		20	21	
Energy intake (kcal/day)	1908.5 ± 710.8	1443.0 ± 364.4	2632.4 ± 875.5	<0.001	1424.5 ± 402.7	2622.0 ± 879.5	<0.001	1694.2 ± 580.2	2341.2 ± 967.5	<0.001	1475.0 ± 527.8	2462.9 ± 852.5	<0.001

Statistical tests: Continuous variables were analyzed using ANOVA or Kruskal–Wallis rank sum test. Categorical variables were analyzed using Chi-square tests.

**Table 4:** Relationship between dietary patterns and metabolic syndrome.

Cluster		Model 1	*p*	Model 2	*p*	Model 3	*p*
		OR, 95% CI		OR, 95% CI		OR, 95% CI	
Sugar–Processed	Q1	ref		ref		ref	
	Q4	2.33 (1.19, 4.57)	0.014	2.24 (1.11, 4.51)	0.025	1.10 (0.42, 2.84)	0.849
Alcohol–Meat	Q1	ref		ref		ref	
	Q4	2.37 (1.17, 4.79)	0.017	2.49 (1.13, 5.51)	0.024	1.29 (0.45, 3.70)	0.635
Legume–Nut	Q1	ref		ref		ref	
	Q4	2.30 (1.09, 4.88)	0.029	2.48 (1.14, 5.40)	0.022	2.63 (1.08, 6.37)	0.033
Egg–Vegetable	Q1	ref		ref		ref	
	Q4	1.07 (0.55, 2.12)	0.837	1.22 (0.57, 2.64)	0.608	0.26 (0.10, 0.70)	0.007

Statistical test: analyzed using logistic regression models.

## Data Availability

The original contributions presented in the study are included in the article/Supplementary Materials, further inquiries can be directed to the corresponding author.

## Supplementary Materials

The following supporting information can be downloaded at: https://www.mdpi.com/article/10.3390/nu16172890/s1, Table S1: KMO and Bartlett’s Test of Sphericity; Table S2: Eigenvalues and Variance Explained by Each Component in Principal Component; Table S3: Description of the food items included in the food categories; Figure S1: Appropriateness of Factor Analysis Scree Plot.
