# Supplementary material for: Impact of Dietary Patterns on Metabolic Syndrome in Young Adults: A Cross-Sectional Study
Source: Nutrients. 2024 Aug 29;16(17):2890. doi: 10.3390/nu16172890 (PMC11397102; doi:10.3390/nu16172890)
Supplement: Supplementary file 1 [file nutrients-16-02890-s001.zip › nutrients-3111591-supplementary.pdf]

Supplementary Materials

Impact of Dietary Patterns on Metabolic Syndrome in Young Adults:

A Cross-Sectional Study

Contents

1 KMO and Bartlett's Test of Sphericity 3

2 Eigenvalues and Variance Explained by Each Component in Principal Component 4

3 Description of the food items included in the food categories 5

4 Appropriateness of Factor Analysis Scree Plot 6

1 KMO and Bartlett's Test of Sphericity

Table S1 KMO and Bartlett's Test of Sphericity

KMO Sample Adequacy		0.771
Bartlett's Test Statistic	Approximate Chi-Square	1941.326
	Degrees of Freedom	136
	Significance	0.000

2 Eigenvalues and Variance Explained by Each Component in Principal Component

Table S2 Eigenvalues and Variance Explained by Each Component in Principal Component

Component	Initial Eigenvalues			Sum of Squared Loadings Extracted			Sum of Squared Loadings Rotated		
	Total	Percentage of Variance	Cumulative %	Total	Percentage of Variance	Cumulative %	Total	Percentage of Variance	Cumulative %
1	3.827	22.512	22.512	3.827	22.512	22.512	2.924	17.201	17.201
2	2.424	14.261	36.773	2.424	14.261	36.773	2.215	13.032	30.233
3	1.679	9.875	46.648	1.679	9.875	46.648	2.178	12.810	43.043
4	1.327	7.809	54.457	1.327	7.809	54.457	1.940	11.414	54.457
5	1.070	6.293	60.750						
6	0.877	5.157	65.907						
7	0.763	4.486	70.394						
8	0.731	4.299	74.693						
9	0.621	3.653	78.345						
10	0.611	3.592	81.937						
11	0.541	3.182	85.120						
12	0.507	2.985	88.104						
13	0.481	2.830	90.934						
14	0.435	2.557	93.492						
15	0.417	2.451	95.942						
16	0.389	2.289	98.231						
17	0.301	1.769	100						

Extraction Method: Principal Component Analysis.

3 Description of the food items included in the food categories

Table S3 Description of the food items included in the food categories

Food categories	Food items
Whole Grains(9)	Brown rice, oats, whole wheat bread, whole wheat flour, quinoa, barley, millet, black rice, and corn.
Tubers(6)	Potatoes, sweet potatoes, yams, taro, cassava, and Jerusalem artichokes
Beans and Bean Products(9)	Soybeans, black beans, chickpeas, lentils, tofu, tempeh, soy milk, edamame, and natto
Nuts(7)	Walnut (dried) / Wild walnut (dried), Chestnut (roasted), Pine-nut (roasted) / Almond kernel (roasted) / Cashew (roasted), Peanut, Sunflower seed (roasted) / Pumpkin seed (roasted) / Watermelon seed (roasted), Lotus seed (dried), Sesame (white and black)
Fresh Vegetables(46)	Radish (white / red / green), Carrot (red / yellow), Cowpea / Snow pea / Kidney bean, Pale green soybean, Soybean sprouts, Sprout (Mung bean), Eggplant, Tomato, Okra, Chinese wax gourd, Calabash, Cucumber, Balsam pear, Pumpkin, Scallion, Chinese chive, Chives flowering stalk, Bok choi, Chinese cabbage, Cabbage, Cauliflower, Spinach, Celery, Endive lettuce, Coriander leaf, Amaranth, Chrysanthemum crown daisy, Shepherd's purse, Lettuce stem, Water spinach, Seedling, Bamboo shoot, Daylily flower, Asparagus stem, Lotus root, Water bamboo, Yam, Taro, Straw mushroom / Button mushroom, Gold needle mushroom, Oyster mushroom, Wood ear fungus, Shitake mushroom, Silver ear fungus, Kelp, Laver
Fruits(28)	Apple, Pear, Peach, Plum, Apricot, Date / Date (dried), Cherry, Grape / Raisin, Persimmon, Mulberry, Chinese kiwi fruit, Strawberry, Kumquat (oval), Pomelo, Pineapple, Jackfruit flesh, Longan / Longan (dried), Jujube, Mango, Papaya, Banana, Bayberry, Coconut, Loquat, Casaba / Hami cantaloupe, Watermelon, Durian, Dragon fruit
White Meat(12)	Chicken, Chicken (wing) / Duck (wing), Chicken (leg), Chicken (feet) / Duck (feet), Chicken (liver / heart / gizzard), Chicken (blood) / duck (blood), Duck, Duck (intestine / liver / gizzard), Duck (tongue), Goose, Goose (liver / gizzard), Pigeon / Quail
Red Meat(12)	Pork, Pork (heart / liver / kidney / large intestine), Pork (ear), Pork (hoof), Pork (blood), Beef, Beef (liver/tripe), Beef (tendon), Beef (tongue), Beef (dried), Lamb, Lamb (tripe / liver / large intestine)
Processed Meats(6)	Sausages, ham, bacon, hot dogs, salami, and pastrami
Aquatic and Seafood(15)	Crucian carp, Grass carp / Black carp, Rice eel, Snakehead, Sliver carp, Goldfish carp / Common carp / Bream, Chinese perch, Yellow croaker (large / small), Abalone, Razor clam, Oyster, Scallop (fresh) / Clam, Sea cucumber (fresh), Squid (fresh), Octopus
Milk and Dairy Products(5)	Milk, Whole milk powder, Full fat, Cheese, Cream / Butter / Canned
Eggs(2)	Hen's egg, Goose's egg / Duck's egg / Quail's egg
Cooking Oils(7)	Olive oil, canola oil, sunflower oil, peanut oil, soybean oil, coconut oil, and sesame oil
Fried Foods(6)	Fried chicken, French fries, donuts, tempura, fish and chips, and spring rolls
Sugary Beverages(5)	Soda (e.g., cola, sprite), fruit juices, energy drinks, sweetened iced tea, and sports drinks
Alcoholic Beverages(6)	Beer, wine (red, white), spirits (vodka, whiskey), cocktails, sake, and brandy
Pastries(8)	Cakes, cookies, pies, donuts, tarts, muffins, croissants, and pastries

4 Appropriateness of Factor Analysis Scree Plot

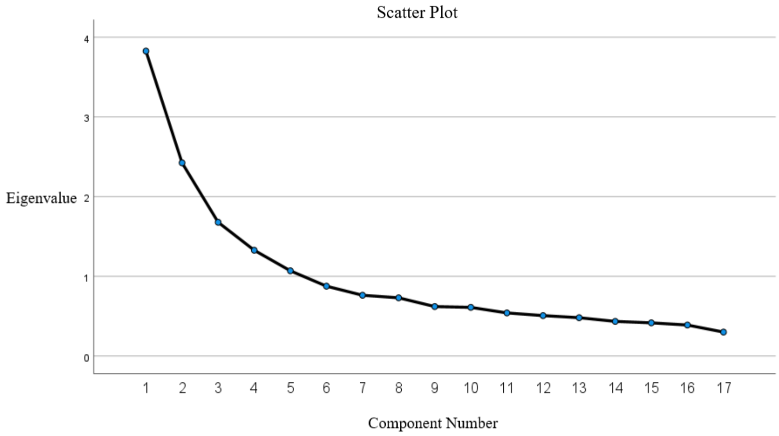

Figure S1 Appropriateness of Factor Analysis Scree Plot
